# Can BPA Analogs Affect Cellular and Biochemical Responses in the Microalga *Phaeodactylum tricornutum* Bohlin?

**DOI:** 10.3390/jox13030030

**Published:** 2023-09-08

**Authors:** Jacopo Fabrello, Maria Ciscato, Emanuela Moschin, Francesca Dalla Vecchia, Isabella Moro, Valerio Matozzo

**Affiliations:** Department of Biology, University of Padova, Via Basssi 58/B, 35131 Padova, Italy; jacopo.fabrello@studenti.unipd.it (J.F.); mariaciscato95@gmail.com (M.C.); emanuela.moschin@unipd.it (E.M.); francesca.dallavecchia@unipd.it (F.D.V.); isabella.moro@unipd.it (I.M.)

**Keywords:** bisphenol analogs, BPA, microalgae, biomarkers, *Phaeodactylum tricornutum*

## Abstract

Bisphenol A analogs (BPA analogs) are emerging contaminants with a rising production caused by the replacement of BPA with these compounds. The increased production of BPA analogs is leading to their increased release into various ecosystems, including marine ones. The aim of this study was to evaluate the biological effects of BPA analogs on a primary producer, the diatom *Phaeodactylum tricornutum* Bohlin. Three different BPA analogs (BPAF, BPF, and BPS) and their mixture were tested at the environmental relevant concentration of 300 ng/L. Growth, cell size and several biomarkers of oxidative stress and oxidative damage were measured. Our results indicated that the tested compounds caused a reduced growth rate and induced oxidative stress, altering many antioxidant enzymes in *P. tricornutum*. However, no oxidative damages were observed.

## 1. Introduction

**Figure 1:**
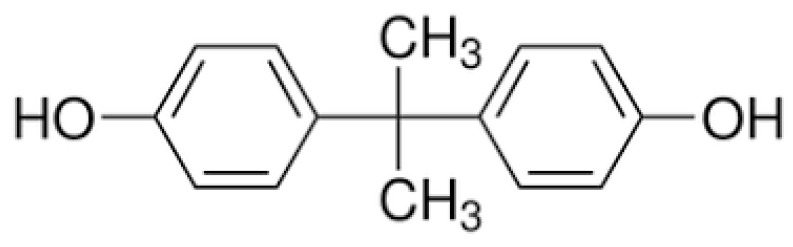
Structural formula of bisphenol A.

Among the emerging contaminants, bisphenols have been the subject of much interest by researchers. The main bisphenol is bisphenol A (BPA), which is used as a plasticizer in polycarbonate plastic production (Figure 1).

**Figure 2:**
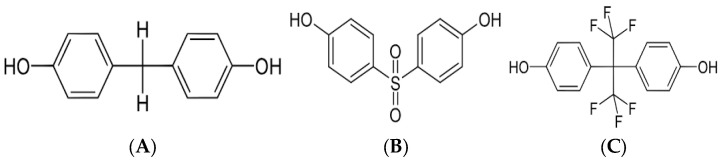
Structural formula of bisphenol F (**A**), bisphenol S (**B**), and bisphenol AF (**C**).

BPA has many other uses, such as in thermal paper as a color developer, in epoxy resins, in medical equipment, and in consumer electronics [1]. Recently, BPA has been restricted in some industrial activities. Indeed, it is known that it can cause oxidative stress and toxic effects on the reproductive system in both humans and animals [2,3]. The main restrictions regard the ban of BPA in the manufacturing of baby bottles in the USA, Canada, the EU, and Australia. In addition, the BPA usage was settled to less than 0.02% in thermal paper in the EU [4]. These restrictions have led to the replacement of BPA with other bisphenols called bisphenol A analogs (BPA analogs). The main BPA analogs that are currently used are bisphenol F (BPF), bisphenol S (BPS), and bisphenol AF (BPAF) (Figure 2A–C). These compounds have many uses, such as in food packaging, water pipes, industrial floors, plastics, adhesives, epoxy glues, thermal receipt papers, and in common polymer applications [3]. Besides their increasing use, data on the production of BPA analogs are scarce, even if many studies have shown that it is increasing [5,6]. Indeed, BPS production increased twofold between 2016 and 2017, with a reported annual production of 1000–10,000 tons [7,8]. Similarly, the annual production of BPAF significantly increased between 1986 and 2002 in the USA, and its manufacturing/import in the EU yields 100–1000 tons per year [9,10].

Bisphenols reach the aquatic environment from discharges from urban and industrial areas, as well as by plastic degradation. Indeed, due to their use as plasticizers, they are released by microplastics that can also transfer these compounds into other environments. Bisphenols are mainly biodegraded, even if their half-lives in water are highly variable due to the environmental conditions. However, some bisphenols can be rapidly degraded in river water, such as BPF, which was completely biodegraded in 37 days, while others (BPAF, BPE, and BPB) are more persistent [11]. The EU settled guidelines to classify the toxicity of a compound according to the EC_50_ value: very toxic to aquatic organisms (<1 mg/L), toxic to aquatic organisms (1–10 mg/L), harmful to aquatic organisms (10–100 mg/L); compounds with an associated EC_50_ value above 100 mg/L are not classified [12]. In many studies BPS has been classified as harmful for the different microalgal species tested with an EC_50_ after 96 h of 85.48 mg/L [13,14], while BPAF was considered as toxic with an EC_50_ value of 1.78 mg/L and 3 mg/L after 96 and 72 h, respectively [14,15]. However, some studies on other algae have reported that BPS is more toxic whereas BPAF showed a lower toxicity [16,17]. Regarding the BPF, its toxic classification varies from toxic to harmful for aquatic organisms, with reported EC_50_ values that range from 9.2 mg/L to 12.3 mg/L [18,19]. The environmental concentrations of BPA analogs are often in the range of a few ng/L [20]. However, recent studies have indicated higher concentrations, with levels of hundreds and thousands of ng/L in both fresh and marine environments [20]. These values exceed the predicted no-effect concentration (PNEC) of BPA settled by the EU at 1500 ng/L for freshwater and 150 ng/L in marine water [21]. For instance, BPAF was recorded at a mean concentration of 140 ng/L in freshwater in China [22], while BPF reached 2850 ng/L and BPS reached 65,600 ng/L in the freshwater of Japan and China, respectively [23,24]. In the marine environment, bisphenol analogs have a common concentration from very few ng/L up to tens of ng/L, even if they reach higher concentrations, as in the case of BPF, which had maximum concentrations of 282 ng/L and 1470 ng/L in seawater in South China and in the Tokyo Bay, respectively [24,25]. The high interest and concern regarding BPA analogs, along with the consequences of their rising production/release, collides with the limited data available on their toxicity to microalgae and primary producers in general. In this study, for the first time, we evaluated the effects of single BPAF, BPF, BPS, and their mixture at environmentally realistic concentrations on the growth, cell size, and biochemical parameters of the microalga *Phaeodactylum tricornutum* Bohlin 1897, a diatom taxon commonly present in seawater as a primary producer.

## 2. Materials and Methods

### 2.1. Microalga Exposure

*P. tricornutum* was purchased from the Culture Collection of Algae at Goettingen University (SAG) and was grown in the F/2 medium [26]. The stock culture was acclimated to the experimental conditions in a culture chamber at 16 °C, a light intensity of 40.5 µmol photons m^−2^s^−1^, and a photoperiod of 12:12 light/dark. The stock solutions (1 mg/L) of BPAF and BPF were made in methanol, while the BPS was dissolved in distilled water. Stocks of microalgal cultures were inoculated in Erlenmeyer flasks containing 200 mL of F/2 medium and exposed in triplicate to the bisphenols BPAF, BPF, and BPS, as well as to their mixture. A concentration of 300 ng/L of each bisphenol or 100 ng/L of each of them in the mixture treatment was adopted. In addition, a control plus methanol was tested. Cells from the stock culture (*inoculum*) were added to obtain an initial concentration of 5 × 10^5^ cells/mL. The cell concentration was measured through a Scepter™ 2.0 Automated Cell Counter (Millipore, Berlington, MA, USA); the data had previously been validated using a Neubauer hemocytometer through a light microscope (Leica, Wetzlar, Germany). All the biochemical analyses were carried out in two different growth phases: exponential and at the beginning of the stationary phase, corresponding to 5 and 9 days of exposure, respectively.

### 2.2. Growth Curve and Growth Rate

Microalgal growth, diameter, and volume were measured using a Scepter™ 2.0 Automated Cell Counter (Millipore, Berlington, MA, USA) every day of exposure. Briefly, 20 µL of cell culture was added to 2 mL of Coulter Isoton II diluent. The diatom concentration was expressed as the number of cells (10^5^)/mL of the medium, while the diameter and volume were expressed in µm and picoliters (pL), respectively. In addition, growth rates were calculated according to the following equation:

$$µ=\ln\;(N_{1}/N_{0})/(t_{1}-t_{0})$$

where $N_{1}$ and $N_{0}$ represent cell concentrations at $t_{1}$ and $t_{0}$ [27].

### 2.3. Biochemical Assays

Culture cells were centrifuged at 6000 g, and the pellets were frozen in liquid nitrogen and stored at −80 °C. Analyses were performed in triplicate. Prior to the biochemical assays, the pellets were sonicated in four volumes of 10 mM Tris-HCl buffer, pH 7.5, containing 0.15 M KCl, 0.5 M sucrose, 1 mM EDTA, and protease inhibitor cocktail (1:10 *v*/*v*) (Merck, Milano, Italy). The protein concentration was quantified according to Bradford et al. [28].

The total antioxidant capacity was measured according to the CUPRAC method [29]. Briefly, the reaction produced cupric ions that reacted with a specific indicator, and the complex was measured at 450 nm using a microplate reader. The results are expressed as mM of Trolox equivalents.

The total superoxide dismutase (SOD) activity was measured according to the method proposed by Crapo et al. [30] using the xanthine oxidase/cytochrome c reaction. Enzyme activity was expressed as U/mg protein, and one unit of SOD was defined as the amount of sample causing 50% inhibition under the assay conditions.

Catalase (CAT) activity was measured following the method proposed by Aebi [31]. The enzyme activity in a volume of 30 µL of tissue SN was measured at 240 nm and expressed as U/mg protein; one unit of CAT was defined as the amount of enzyme that catalyzed the detoxification of 1 μmol of H_2_O_2_/min.

Glutathione reductase (GR) activity was evaluated according to Smith et al. [32], by measuring the (5-thio (2-nitrobenzoic acid)) TNB production at 412 nm. The enzyme activity was expressed as U/mg protein.

Glutathione S-transferase (GST) activity was measured according to the method described by Habig et al. [33], using 1-chloro-2,4-dinitrobenzene (CDNB) and reduced glutathione (GSH) as the substrate. GST activity was expressed as nmol/min/mg protein.

Selenium-dependent glutathione peroxidase (GPX) activity was measured following the method of Lawrence and Burk [34], by measuring the decreased absorbance at 340 nm caused by the NADPH oxidation. GPX Se-dependent activity was expressed as mmoli/min/mg protein.

Ascorbate peroxidase (APX) activity was evaluated according to Nakano and Asada [35] and modified by Janknegt et al. [36] using the ascorbate and hydrogen peroxide as the reactives. APX activity was expressed as U/mg protein.

Oxidative damage was measured in both proteins, using the protein carbonyl content assay (PCC), and in lipids, measuring the level of peroxidation (LPO). Briefly, PCC was measured using the method of Mecocci et al. [37], following the reaction with 2,4-dinitrophenylhydrazide (DNPH). The results were expressed as nmol carbonyl group/mg protein. The LPO was quantified using the malondialdehyde (MDA) assay, according to the method of Buege and Aust [38]. Absorbance was read spectrophotometrically at 532 nm, and the results were expressed as nmoles of thiobarbituric reactive substances (TBARS)/mg protein. TBARS, considered as “MDA-like peroxide products”, were quantified by reference to MDA absorbance (ε = 156 × 103 M^−1^ cm^−1^) [39].

### 2.4. Statistical Analysis

The effects of the independent factors, namely “treatment”, “growth phase”, and their interaction (treatment * growth phase), were evaluated by means of a two-way ANOVA analysis for each biomarker, using OriginPro software (OriginPro, Version 2022. OriginLab Corporation, Northampton, MA, USA). The post hoc test (Fisher’s test) was performed for pairwise comparisons. All results are expressed as the mean ± standard deviation (SD).

## 3. Results

**Figure 3:**
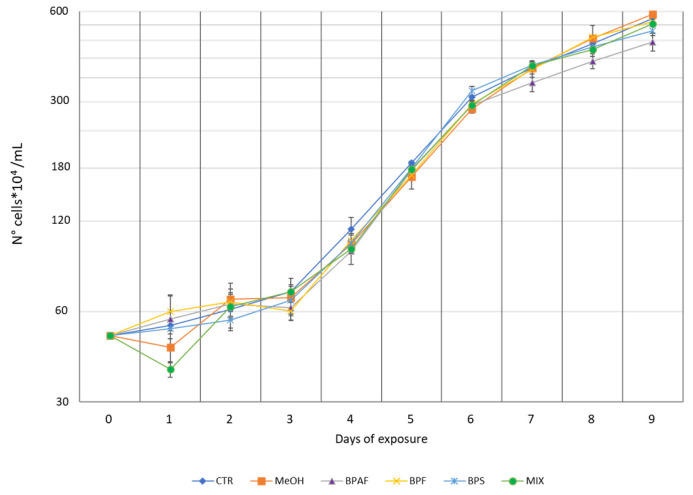
Growth of *P. tricornutum*, expressed as number of cells × $10^{4}$/mL, during exposure to bisphenols BPAF, BPF, and BPS, as well as to their mixture (MIX). Control is reported as CTR; control + solvent (methanol) is reported as MeOH. Each point is the mean of three replicates ± SD.

During the exposure to different bisphenols, the growth curve was altered by the factors of “growth phase”, “treatment”, and their interaction (*p* < 0.001) (Table S1 in Supplementary Materials). The bisphenols caused a general reduction in growth during the exponential phase (4–6 days); however, the post hoc test revealed that only BPAF caused a significant decrease in microalgal growth after 7 and 8 days of exposure. In detail, we recorded growth inhibition percentages of 10.86% and 12.66% caused by BPAF after 7 and 8 days, respectively, with respect to the control. Moreover, at the stationary phase (9 days), both BPAF and BPS caused a significant decrease in the growth, with a growth inhibition percentage of 16.37% and 8.7%, respectively (Figure 3) (Table S2 in Supplementary Materials). Interestingly, the mixture did not have any effect on the growth. Moreover, we calculated the growth rates of the six different treatments for each day of exposure (Table S3 in Supplementary Materials), and we also calculated the growth rates during the exponential phase and the stationary phase (Table S4 in Supplementary Materials).

**Figure 4:**
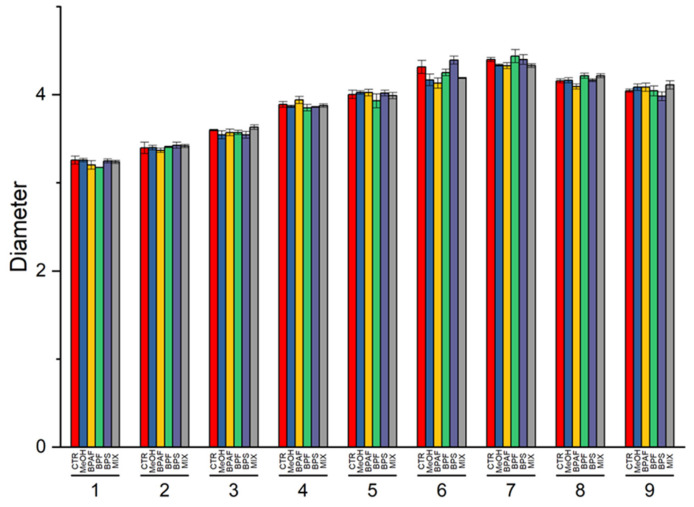
Diameter values of cells, expressed as μm, during the nine days of exposure. The values are mean ± SD (*n*  =  3).

**Figure 5:**
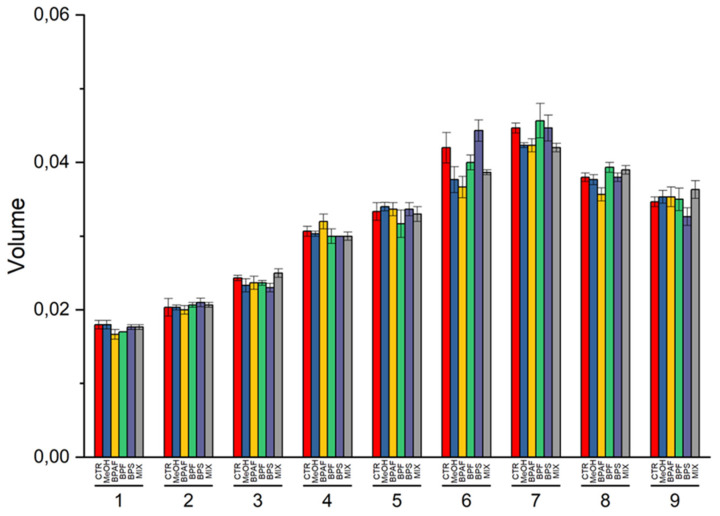
Volume values of cells, expressed as pL (picolitres), during the nine days of exposure. The values are mean ± SD (*n* = 3).

During the exposure, both the mean cell diameter and mean cell volume of the microalgae were measured daily. Both of the two cellular parameters were affected by the “growth phase” factor (*p* < 0.001) and its interaction with “treatment” (*p* < 0.05 and *p* < 0.01, respectively) (Table S1). The post hoc test revealed that both BPAF and MIX caused a significant decrease in cell diameter and volume after 6 days of exposure (*p* < 0.001 and *p* < 0.05, respectively) (Tables S5 and S6 in Supplementary Materials; Figure 4 and Figure 5).

**Figure 6:**
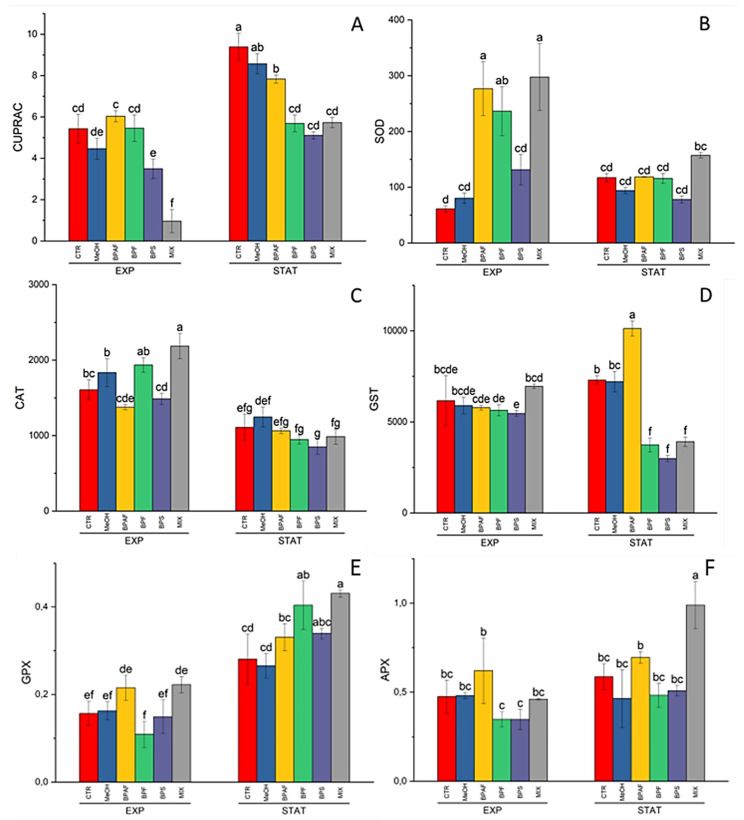
CUPRAC levels, expressed as mM of Trolox equivalents/mg protein (**A**); SOD activity, expressed as U SOD/mg protein (**B**); CAT activity, expressed as U CAT/mg protein (**C**); GST activity, expressed as nmol/min/mg protein (**D**); GPX activity, expressed as U GPX/mg protein (**E**); and APX activity, expressed as U APX/mg protein (**F**) in the exponential (EXP) and stationary (STAT) growth phases. The values are mean ± SD (*n* = 3). Different letters indicate statistically significant differences in comparison with the related control (*p* < 0.05).

The total antioxidant capacity, measured using the CUPRAC assay, was significantly altered by the factors “growth phase” and “treatment”, as well as their interaction (*p* < 0.001) (Table S7 in Supplementary Materials). In addition, the post hoc test revealed a significant reduction in the total antioxidant capacity with both BPS and MIX treatment during the exponential phase (Figure 6A). Similarly, a significant reduction in total antioxidant capacity was observed in all the bisphenol treatments during the stationary phase, with respect to the controls (Figure 6A).

As for SOD and CAT activity, the independent factors (growth phase, treatment, and their interaction) exerted effects on enzyme activities. In detail, the two-way ANOVA demonstrated that the factors “growth phase” and “treatment” significantly affected (*p* < 0.001) SOD activity, as well as their interaction (*p* < 0.01) (Table S7 in Supplementary Materials). CAT activity was influenced by growth phase (*p* < 0.001), treatment (*p* < 0.01), and their interaction (*p* < 0.05) (Table S7 in Supplementary Materials). In detail, SOD activity was significantly increased after 5 days of exposure to BPAF, BPF, and MIX, while no differences in SOD activity were observed after 9 days of exposure (Figure 6B). As for CAT activity, exposure to MIX increased the enzyme activity during the exponential phase, whereas no differences were recorded during the stationary phase (Figure 6C).

Regarding the glutathione-related enzymes, GR activity was influenced only by the factor “growth phase” (Table S7 and Figure S1 in Supplementary Materials), while GST activity was altered by both “treatment” and its interaction with “growth phase” (*p* < 0.001) (Table S7 in Supplementary Materials). Moreover, GST activity was significantly affected only during the stationary phase, with a marked increase in the BPAF-treated group, followed by a significant reduction in the BPF-, BPS-, and MIX-treated groups (Figure 6D).

The two peroxidase enzymes, GPX and APX, were altered by the factors “growth phase” (*p* < 0.001 and *p* < 0.01, respectively) and “treatment” (*p* < 0.05) (Table S8 in Supplementary Materials). In detail, the GPX activity was significantly increased in the stationary phase by exposure to BPF and MIX (Figure 6E), while the ascorbate peroxidase was increased by exposure to MIX after 9 days of treatment (Figure 6F).

Lastly, statistical analysis indicated that LPO was altered only by the factor “growth phase” (*p* < 0.01) (Table S8 and Figure S2A in Supplementary Materials), while the PCC value was not affected by any of the independent factors (Table S8 and Figure S2B in Supplementary Materials).

## 4. Discussion

### 4.1. Growth and Cell Size

In this study, we tested the effects of three of the main BPA analogs (BPAF, BPF, and BPS) and their mixture on the diatom *P. tricornutum*. The growth curves were mainly affected by BPAF during the last phase of exposure. In addition, the BPS also caused a reduction in the cell density at day 9. The BPF did not cause any effect, while with the MIX treatment, a general reduction in growth was observed, even if it was non-significant. Indeed, the absence of a significant reduction in cell density values and a contemporary significant effect observed in the BPAF treatment suggest a sort of antagonistic interaction between the three BPA analogs in the MIX treatment. In addition, we speculated that the BPAF concentration was too low to induce a significant reduction in cell density. Other studies indicate that many algal species are very tolerant to BPA exposure in term of cell growth. Indeed, the EC_50_ values are in the order of mg/L [40], even if some species can be more sensitive [41]. Our results are in accordance with those of Ding et al. [16], which reported no significant effects on the growth rate of *Chlorella vulgaris* after exposure to environmentally relevant concentrations (≤0.5 mg/L) of BPS and BPA, even if the growth decreased at higher concentrations. In other studies, the tested concentrations showed effects on the growth curve; however, the tested concentrations were higher than the environmental levels. Indeed, Tišler et al. [15] recorded a value of 50% inhibition of growth in *Desmodesmus subspicatus* after 72 h of exposure to both BPA and BPF (22.1 and 19.6 mg/L, respectively). In that study, BPAF showed a 50% inhibition of growth at 3.0 mg/L, which is 1000 times higher than the concentration tested in the present study. A summary of studies on the effects of bisphenols on the growth of microalgae is reported in Table 1.

We observed that both the cell diameter and cell volume were altered by the “growth phase” factor and its interaction with “treatment”. In detail, both diameter and volume increased during the experiment and reached the highest values after seven days of exposure; then, they decreased. Interestingly, both BPAF and MIX caused a significant decrease in both cell diameter and volume after 6 days of exposure. Recently, Li et al. [43] observed that bisphenols caused a decrease in the cell size of *Tetrahymena thermophila*. Indeed, BPA (2.6 and 13.0 μM), BPAF (13.0 μM), BPB (13.0 μM), and BPS (13.0 μM) caused a significant reduction in cell volume after 12 h of exposure in the adaptive growth phase, and this trend was maintained after 30 h of exposure. Furthermore, after 60 h of exposure in the stable growth phase, the cell volume was reduced in all the treatments, except for the BPE at a concentration of 2.6 μM [43]. *P. tricornutum* was used in another study to evaluate the effects of six different bisphenols BPA, BPS, BPAP, BPAF, BPFL, and BPC were tested at various concentrations, namely, 5, 20, 40, 80, 150, and 300 μM [47]. The authors observed that the growth of this microalga was inhibited by all the concentrations at all times of exposure, except for bisphenol C at 5 μM and 72 h. The authors ranked the toxicity levels of the bisphenols to this species as follows: BPC < BPA < BPS < BPFL ≈ BPAF ≈ BPAP after 72 h of exposure. In the same study, the authors also evaluated the effects of bisphenols on two other species of microalgae: *Tetraselmis suecica*, which was the most sensitive species, and *Nannochloropsis gaditana*, which was the species most resistant to bisphenols, even if some effects have been observed on this species as well [47]. Interestingly, the authors observed that almost all of the tested bisphenols caused reductions in the cell volume of *P*. *tricornutum*, apart from BPS, which provoked a significant increase in this parameter at the highest concentrations, and BPFL, which did not cause any effect. On the contrary, *T. suecica* cells showed a significant increase in cell size, mainly at higher concentrations and after 72 h of incubation, while *N*. *gaditana* was less affected than the other two species.

### 4.2. Biomarker Responses

In the present study, several biomarkers indicative of oxidative stress were measured in *P. tricornutum* treated with bisphenol analogs. Regarding the total antioxidant capacity, a reduction was observed in the microalga exposed to BPS and MIX for five days. Moreover, the same effect was also observed in the microalga after nine days of exposure to BPAF, BPF, BPS, and MIX. The SOD activity was significantly increased by BPAF, BPF, and MIX exposure during the exponential growth phase. However, in the stationary phase, all treatments caused effects. Similarly, in the case of CAT activity, it was significantly increased only by MIX after five days, while during the stationary phase, there were no alterations. To our knowledge, our study adopted the lowest concentrations which have been tested in the literature. Indeed, many other studies observed alterations to the antioxidant system, but with very high concentrations of bisphenols, mainly BPA. However, our observations of an impairment in the microalgal antioxidant system are in accordance with the bisphenols’ capacity to produce ROS, which has been reported in many studies. Indeed, recently, *P. tricornutum* showed an increase in ROS production for most of the tested bisphenols (BPC, BPS, BPA, BPAF, BPAP, and BPFL) over 72 h at different dosages (5, 20, 40, 80, 150 and 300 μM). In detail, BPAP showed the highest ROS production, followed by BPFL > BPAF > BPA > BPS > BPC [47]. Similarly, in *Chlorella pyrenoidosa*, BPA caused ROS increases at concentrations of 5, 8, 11, and 15 mg/L, while BPS caused an increase in ROS levels at higher concentrations, such as 15, 20, and 40 mg/L. Moreover, the mixture of the two bisphenols at a ratio of 1:1 caused increased ROS only at the two lowest concentrations [13]. Recently, the activities of both SOD and CAT were measured in two freshwater algae, *Chlorella vulgaris* and *Navicula* sp., which had been exposed to BPS [48]. The authors observed that in *C. vulgaris,* the SOD activity increased significantly at 1, 5, 10, 20, and 50 mg/L of BPS, while CAT activity increased at 20 and 100 mg/L of BPS. In an opposite way, in *Navicula* sp., there was a significant increase in the activity of these two enzymes at 1 mg/L only [48]. Similarly, after 4 and 8 days of exposure, all the tested concentrations of BPA (4, 6, 8, 10, and 12 mg/L) increased the SOD activity in *Cyclotella caspia*. However, such an increment, with respect to the control, was lower after 12, 16, and 20 days of exposure [49]. Interestingly, all of the tested concentrations caused an increase in the activity of both SOD and CAT after 96 h of exposure of *C. pyrenoidosa* to BPA [45]. In another study with the cyanobacterium *Microcystis aeruginosa,* the authors observed that SOD activity was not altered by BPA at a concentration range of 0.1–1.0 μM. However, higher concentrations (5−50 μM) caused a significant increase in SOD activity. Moreover, the authors observed that the GSH content was significantly increased by the BPA-tested concentrations after 3, 9, and 15 days of exposure [50]. The effects of another bisphenol analog, namely, bisphenol BHPF, were tested in the microalga *Chlorella vulgaris* [51]. Contrary to our results, the authors found that SOD activity significantly decreased at 1, 10, and 20 mg/L of BHPF, while CAT activity remained unchanged after 72 h of exposure. Alterations in both SOD and CAT activity were also observed during chronic exposure. Indeed, with long-term exposure, SOD activity increased after 15 days at 1 mg/L and 10 mg/L of BPA, while after 30 days, only 1 mg/L of BPA had an effect, increasing the SOD activity in *C. pyrenoidosa* [45]. In the case of *Scenedesmus obliquus*, only after 15 days of exposure to 1 mg/L of BPA was there an increase in SOD activity [45]. Moreover, the two algae showed increased CAT activity at 50 mg /L of BPA after 15 and 30 days, and, in the case of *S. obliquus,* the CAT activity also increased at 10 mg/L after 15 days and at 1 mg/L and 10 mg/L of BPA after 30 days of exposure [45]. The GST enzyme is an antioxidant enzyme involved in the detoxification activity of xenobiotics. In our study, GST activity was increased after exposure to BPAF during the stationary phase, while BPF, BPS, and MIX caused a significant reduction in enzyme activity. Conversely, the exposure of *Navicula incerta*—another marine diatom species—to BPA increased both SOD and GST activity in a dose-dependent manner, while the peroxidase activity decreased at 5 mg/L of BPA [52]. In addition, increased activity of both CAT and GST was reported in *Picocystis* sp. exposed to 25, 50, and 75 mg/L of BPA [53]. As for GR, which is an enzyme involved in the glutathione cycle, we did not observe any effects of the tested compounds during exposure. On the contrary, Jiefeng et al. [54] observed a reduction in SOD, CAT, peroxidase, and GR activity at all the BPA treatments (1, 5, 20, and 50 mg/L), with a contemporary general increase in GSH levels. However, much higher concentrations were tested in that study. Moreover, the authors observed that 20 mg/L and 50 mg/L of BPA caused significant reductions in the total antioxidant capacity (Jiefeng et al. [54]). Another two important enzymes that we assessed are glutathione peroxidase (GPX) and ascorbate peroxidase (APX). These two enzymes are fundamental antioxidant enzymes. Indeed, they detoxify hydrogen peroxide, an important ROS species, using glutathione or ascorbate, respectively. The GPX activity was increased after exposure to BPF and MIX during the stationary phase, while APX increased after nine days of exposure to MIX. Similarly, APX activity was increased by BPA exposure in the microalga *Picocystis* sp. when it was treated for 1, 2, 3, 4, and 5 days with 25, 50, and 75 mg/L; in addition, after 4 and 5 days, the APX activity was also increased by 10 mg/L of BPA. However, at 1 mg/L of BPA, no alterations in APX activity were recorded [53]. In the latter study, the activity of APX measured in *Graesiella* sp. showed an opposite pattern of variation with respect to that of *Picocystis* sp., with decreased enzyme activity at higher BPA concentrations [53].

Oxidative damage to macromolecules is caused by an excessive presence of ROS species that are not efficiently removed by the antioxidant system. The oxidative damage can be evaluated by measuring the levels of carbonyl groups in proteins using a PCC assay and the level of lipid peroxidation through an LPO assay. Based on the results of PCC and LPO assays, we can exclude the notion that bisphenol analogs can induce oxidative damage in *P. tricornutum*, at least under the experimental conditions adopted in our study. However, other studies have reported that bisphenols can cause oxidative damage [40]. For instance, Yang et al. [50] reported oxidative damage to lipids (increased MDA level) after 3, 9, and 15 days of exposure of the cyanobacterium *M. aeruginosa* to 0.1, 1, 5, 10, and 50 mg/L of BPA. However, the MDA levels decreased during the time course [50]. BPA was able to increase the MDA levels at doses above 5 mg/L in both the green alga *Scenedesmus quadricauda* and the cyanobacterium *Cylindrospermopsis raciborskii*, even if there were decreased MDA levels in the cyanobacterium at a concentration of 20 mg/L [55]. In addition, in *C. vulgaris*, all of the tested concentrations of BPS increased the MDA content, while in *Navicula* sp., there was an increased MDA level only at the two highest tested concentrations (50 mg/L and 100 mg/L) [48]. In this study, our results on the effects of MIX agree with those of previous studies [12,15]. Indeed, a higher toxicity of the mixture than that of the single compounds was recorded, with a synergistic effect between them. This result reveals that the antioxidant systems of marine microalga can be markedly affected by BPA analogs, which may be present in aquatic environments either individually or as mixtures. As for cell growth, we hypothesized that antagonist interactions between the tested BPA analogs occurred during the MIX treatment. This condition did not cause significant cell density reduction when compared to the BPAF treatment.

## 5. Conclusions

In the present study, we observed that bisphenol analogs, at environmentally realistic concentrations, affected the growth and the cell size of the diatom species *P. tricornutum*. In addition, we observed alterations to the antioxidant systems, with some antioxidant enzyme activities being affected. We observed that the mixture caused higher alterations to the measured biochemical parameters in comparison to the single bisphenols. In conclusion, the bisphenol A analogs tested in this survey can be harmful for the microalga *P. tricornutum*, as previously observed for BPA. Studies on the molecular interactions of bisphenols and their impact on the ultrastructures of microalga are needed in order to better determine the ecotoxicological profiles of these compounds.

## Figures and Tables

**Table 1:** Effects of bisphenols on the growth of microalgae. The following information is reported: compound, species, concentration (reports all the tested concentrations), effects on growth (reports the concentration used, the time of exposure, and the effect), time (reports the total time of exposure), and Ref.

Compound	Species	Concentration	Effects on Growth	Time	Ref.
BPA	*Chlorella pyrenoidosa*	2, 5, 8, 11, 15 mg/L	2 mg/L (1, 2, 3 d): increased 2 mg/L (4, 5, 6 d): inhibition 5, 8, 11, 15 mg/L: inhibition	6 days	[13]
BPS	*C. pyrenoidosa*	5, 10, 15, 20, 40 mg/L	5 mg/L (1, 2, 3, 4 d): inhibition 5 mg/L (5, 6 d): increased 10, 15, 20, 40 mg/L: inhibition	6 days	[13]
BPA + BPS	*C. pyrenoidosa*	0.05 P, 0.1 P, 0.2 P, 0.3 P, 0.4 P, 0.5 P	0.05 P (1, 5, 6 d): increased 0.1 P (5, 6 d): inhibition 0.2, 0.3, 0.4, 0.5 P: inhibition	6 days	[13]
BPA	*Tetraselmis* sp.	2.34, 4.69, 9.38, 18.75, 37.5, 75, 150, 300 mg/L	Inhibition	24, 48, 72, 96 h	[42]
BPA, BPAF, BPB, BPE	*Tetrahymena thermophila*	2.6–13.0 μM	BPA (13 μM): inhibition BPAF (13 μM): inhibition BPB (2.6–13 μM): inhibition BPE (2.6–13 μM): inhibition	30 h	[43]
BPA	*C. pyrenoidosa*	0.1, 1, 10 mg/L	0.1 mg/L (2–5 d): increased 1 mg/ (4–5 d): inhibition 10 mg/L (1–5 d): inhibition	5 days	[44]
BPA	*C. pyrenoidosa*	1, 5, 10, 25, 50 mg/L	1, 5 mg/L (96 h): inhibition 10 mg/L (72, 96, 144 h): inhibition 25 mg/L (48, 72, 96 h): inhibition 50 mg/L (24, 48, 72, 96 h): inhibition	144 h	[45]
BPA	*Scenedesmus obliquus*	1, 5, 10, 25, 50 mg/L	10 mg/L (24, 96 h): inhibition 25 mg/L (24, 48, 72, 96 h): inhibition 50 mg/L (24, 48, 72, 96 h): inhibition	144 h	[45]
BPA	*C. pyrenoidosa*	1, 10, 50 mg/L	1, 50 mg/L (30 d): increased	30 d	[45]
BPA	*Stephanodiscus hantzschii*	0.01, 0.1, 1, 3, 5, 7, 9 mg/L	≤1 mg/L: no effects 3 mg/L (4 d): inhibition 3 mg/L (8, 12, 16 d): increased 5, 7, 9 mg/L: inhibition	16 d	[46]

## Data Availability

The data presented in this study are available in this article and supplementary materials.

## Supplementary Materials

The following supporting information can be downloaded at: https://www.mdpi.com/article/10.3390/jox13030030/s1. Table S1 Results of the two-way ANOVA analysis performed on cell density, cell diameter and cell volume. Significant values (*p* < 0.05) are in red; Table S2 Results obtained from the post-hoc test performed on the cell density values. Each cell reports the *p* value of the corresponding treatment in comparison to the control of the same day. Significant values (*p* < 0.05) are in red; Table S3 Growth rate (µ) calculated for each of the six treatments during the nine days of exposure; Table S4 Growth rate of exponential phase (µ EXP) and stationary phase (µ STAT) calculated for each of the six treatments; Table S5 Results of the post-hoc test performed on the cell diameter values. Each cell reports the *p* value of the corresponding treatment in comparison to the control of the same day. Significant values (*p* < 0.05) are in red; Table S6 Results of the post-hoc test performed on the cell volume values. Each cell reports the *p* value of the corresponding treatment in comparison to the control of the same day. Significant values (*p* < 0.05) are in red; Table S7 Results of the two-way ANOVA analyses performed on the various biomarkers analyzed, namely the total antioxidant capacity (CUPRAC), superoxide dismutase (SOD), catalase (CAT), glutathione reductase (GR) and glutathione s-transferase (GST). Significant values (*p* < 0.05) are in red; Table S8 Results of the two-way ANOVA analyses performed on the various biomarkers analyzed, namely glutathione peroxidase (GPX), ascorbate peroxidase (APX), protein carbonyl content (PCC), and lipid peroxidation (LPO). Significant values (*p* < 0.05) are in red; Figure S1 glutathione reductase activity (GR), expressed as U/mg protein; the two growth phases are indicated as EXP and STAT. The values are mean ± SD (n = 3). Different letters indicate statistically significant differences in comparison with the related control (*p* < 0.05); Figure S2 protein carbonyl content (PCC), expressed as nmol/mg protein (A), and lipid peroxidation (LPO), expressed as nmol TBARS/mg protein (B); the two growth phases are indicated as EXP and STAT. The values are mean ± SD (n = 3). Different letters indicate statistically significant differences in comparison with the related control (*p* < 0.05).
