# Supplementary material for: Can BPA Analogs Affect Cellular and Biochemical Responses in the Microalga Phaeodactylum tricornutum Bohlin?
Source: J Xenobiot. 2023 Sep 8;13(3):479–91. doi: 10.3390/jox13030030 (PMC10532965; doi:10.3390/jox13030030)
Supplement: Supplementary file 1 [file jox-13-00030-s001.zip › jox-2572231-supplementary.pdf]

Supplementary Materials

Table S1 Results of the two-way ANOVA analysis performed on cell density, cell diameter and cell volume. Significant values ($p < 0.05$) are in red.

	Source of variation	F value	P value
Cell density	Growth phase	3048,19672	1,80588E-123
	Treatment	12,18796	2,25824E-9
	Interaction	4,33003	7,31494E-10
Diameter	Growth phase	641,12607	2,59423E-87
	Treatment	1,16963	0,32887
	Interaction	1,66047	0,02074
Volume	Growth phase	518,05525	1,95787E-82
	Treatment	1,67877	0,14583
	Interaction	1,9279	0,00403

Table S2 Results obtained from the post-hoc test performed on the cell density values. Each cell reports the P value of the corresponding treatment in comparison to the control of the same day. Significant values ($p < 0.05$) are in red.

	MeOH	BPAF	BPF	BPS	MIX
Day 1	0,47845	0,82192	0,60573	0,90718	0,1951
Day 2	0,68017	0,83445	0,76475	0,68058	0,91911
Day 3	0,79083	0,49471	0,41357	0,70505	0,99102
Day 4	0,29864	0,14081	0,44063	0,34555	0,18337
Day 5	0,1097	0,37191	0,16064	0,54006	0,4398
Day 6	0,09556	0,1212	0,16566	0,19546	0,11987
Day 7	0,8704	<0.0001	0,67113	0,52899	0,63267
Day 8	0,10757	<0.0001	0,05465	0,30835	0,07261
Day 9	0,09741	<0.0001	0,29916	<0.0001	0,06117

Table S3 Growth rate (μ) calculated for each of the six treatments during the nine days of exposure

	Day 1	Day 2	Day 3	Day 4	Day 5	Day 6	Day 7	Day 8	Day 9
μ C	0.076867	0.100163	0.111792	0.203694	0.2646	0.304557	0.293422	0.279867	0.269796
μ MeOH	-0.09241	0.138639	0.09646	0.174749	0.24318	0.289551	0.292712	0.284876	0.273604
μ BPAF	0.125084	0.120033	0.070753	0.161496	0.252976	0.29435	0.27699	0.262935	0.249933
μ BPF	0.184237	0.128379	0.061994	0.182571	0.245967	0.295467	0.291566	0.285841	0.267352
μ BPS	0.050945	0.058536	0.089687	0.177583	0.256705	0.312615	0.296131	0.276601	0.259679
μ MIX	-0.25923	0.109916	0.112427	0.165938	0.254588	0.294312	0.295483	0.274022	0.265323

Table S4 Growth rate of exponential phase (μ EXP) and stationary phase (μ STAT) calculated for each of the six treatments.

	C	MeOH	BPAF	BPF	BPS	MIX
μ EXP	0.497322	0.482641	0.517947	0.528941	0.535544	0.476197
μ STAT	0.200273	0.24171	0.161099	0.21112	0.153808	0.207345

Table S5 Results of the post-hoc test performed on the cell diameter values. Each cell reports the P value of the corresponding treatment in comparison to the control of the same day. Significant values ($p < 0.05$) are in red.

	MeOH	BPAF	BPF	BPS	MIX
Day 1	0,99017	0,32226	0,11819	0,86303	0,71168
Day 2	0,97542	0,60926	0,83407	0,57945	0,69796
Day 3	0,32828	0,62659	0,63096	0,32828	0,53407
Day 4	0,68888	0,3531	0,49058	0,59214	0,79107
Day 5	0,68888	0,69796	0,18298	0,79107	0,80057
Day 6	0,10271	8,27387E-4	0,23108	0,15478	0,02108
Day 7	0,24072	0,19528	0,48673	0,98034	0,19952
Day 8	0,9068	0,22636	0,29608	0,9068	0,29326
Day 9	0,46021	0,46395	0,98034	0,24318	0,22403

Table S6 Results of the post-hoc test performed on the cell volume values. Each cell reports the P value of the corresponding treatment in comparison to the control of the same day. Significant values ($p<0.05$) are in red.

	MeOH	BPAF	BPF	BPS	MIX
Day 1	1	0,33309	0,46746	0,80842	0,80842
Day 2	1	0,80842	0,80842	0,62786	0,80842
Day 3	0,46746	0,62786	0,62786	0,33309	0,62786
Day 4	0,80842	0,33309	0,62786	0,62786	0,62786
Day 5	0,62786	0,80842	0,22689	0,80842	0,80842
Day 6	0,14763	1,74218E-4	0,14763	0,09173	0,01672
Day 7	0,09173	0,09173	0,46746	1	0,05443
Day 8	0,80842	0,09173	0,33309	1	0,46746
Day 9	0,62786	0,62786	0,80842	0,14763	0,22689

Table S7 Results of the two-way ANOVA analyses performed on the various biomarkers analyzed, namely the total antioxidant capacity (CUPRAC), superoxide dismutase (SOD), catalase (CAT), glutathione reductase (GR) and glutathione s-transferase (GST). Significant values ($p < 0.05$) are in red.

Biomarker	Source of variation	F value	P value
CUPRAC	Growth phase	99,66145	5,08098E-10
	Treatment	22,26397	2,68696E-8
	Interaction	7,02131	3,6038E-4
SOD	Growth phase	18,15843	2,71766E-4
	Treatment	9,96497	2,96035E-5
	Interaction	5,15396	0,00237
CAT	Growth phase	105,86848	2,80838E-10
	Treatment	4,07111	0,00811
	Interaction	3,84834	0,01058
GR	Growth phase	8,3984	0,0079
	Treatment	1,38849	0,26381
	Interaction	1,59831	0,19866
GST	Growth phase	0,13332	0,71821
	Treatment	15,64025	7,07074E-7
	Interaction	16,22454	5,09996E-7

Table S8 Results of the two-way ANOVA analyses performed on the various biomarkers analyzed, namely glutathione peroxidase (GPX), ascorbate peroxidase (APX), protein carbonyl content (PCC), and lipid peroxidation (LPO). Significant values ($p<0.05$) are in red.

Biomarker	Source of variation	F value	P value
GPX	Growth phase	82,18097	3,2223E-9
	Treatment	3,14146	0,02543
	Interaction	2,49627	0,05896
APX	Growth phase	9,54206	0,00502
	Treatment	3,74008	0,01206
	Interaction	2,06356	0,1055
PCC	Growth phase	7,97379	0,00939
	Treatment	2,00518	0,11419
	Interaction	1,39022	0,2632
LPO	Growth phase	1,54145	0,2264
	Treatment	1,40571	0,25778
	Interaction	2,00846	0,11369

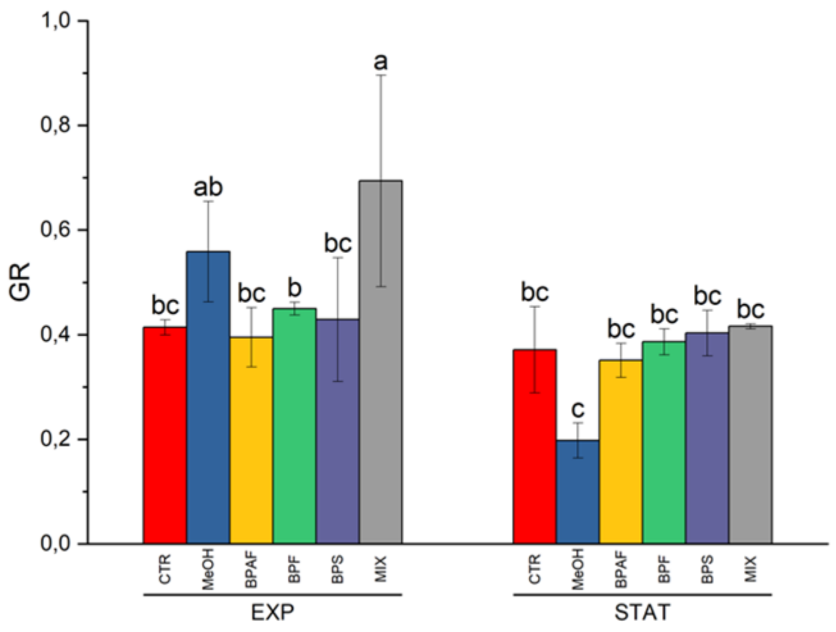

Figure S1 glutathione reductase activity (GR), expressed as U/mg protein; the two growth phases are indicated as EXP and STAT. The values are mean ± SD (n = 3). Different letters indicate statistically significant differences in comparison with the related control ($p < 0.05$).

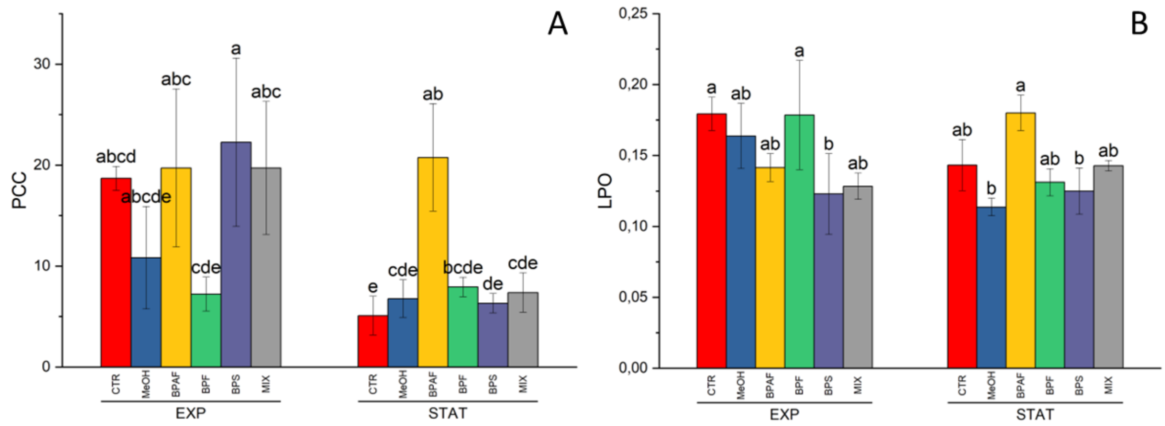

Figure S2 protein carbonyl content (PCC), expressed as nmol/mg protein (A), and lipid peroxidation (LPO), expressed as nmol TBARS/mg protein (B); the two growth phases are indicated as EXP and STAT. The values are mean ± SD (n = 3). Different letters indicate statistically significant differences in comparison with the related control ($p < 0.05$).
